# Inhibition of PI3K increases oxaliplatin sensitivity in cholangiocarcinoma cells

**DOI:** 10.1186/1475-2867-9-3

**Published:** 2009-01-08

**Authors:** Kawin Leelawat, Siriluck Narong, Wandee Udomchaiprasertkul, Surang Leelawat, Sumalee Tungpradubkul

**Affiliations:** 1 Department of Surgery, Rajavithi Hospital, Bangkok, Thailand; 2 Cancer Molecular Therapeutics Unit, Chulabhorn Cancer Center, Chulabhorn Research Institute, Bangkok, Thailand; 3 Faculty of Pharmacy, Rangsit University, Bangkok, Thailand; 4 Department of Biochemistry, Faculty of Science, Mahidol University, Bangkok, Thailand

## Abstract

**Background:**

Resistance of cholangiocarcinoma to chemotherapy is a major problem in cancer treatment. The mechanism of resistance is believed to involve phosphoinositide-3- kinase (PI3K)/Akt activation. Although the platinum-containing compound oxaliplatin has been extensively used in the treatment of several solid tumors, recent data regarding its use to treat cholangiocarcinoma are ambiguous. Oxaliplatin resistance in this disease could potentially involve PI3K pathways. We, therefore, examined the effects of PI3K pathways in cholangiocarcinoma cells in modulating oxaliplatin resistance.

**Results:**

After exposing the cholangiocarcinoma cell lines RMCCA1 and KKU100 to oxaliplatin, the levels of Akt and mTOR phosphorylation increased, as shown by western blot analysis. The WST-1 cell proliferation assay showed increased inhibition of cell growth under high concentrations of oxaliplatin. The combination of oxaliplatin with LY294002, an inhibitor of PI3K, resulted in a remarkable arrest of cell proliferation. Deactivation of mTOR by RAD001 was also synergistic with oxaliplatin, although to a lesser extent. The combination of oxaliplatin and a PI3K inhibitor also resulted in a significant induction of apoptosis, as demonstrated by the TUNEL assay.

**Conclusion:**

Activation of PI3K might protect cholangiocarcinoma cells from oxaliplatininduced cytotoxicity. Although the inhibition of PI3K and the inhibition of mTOR both enhance oxaliplatin-induced cytotoxicity, PI3K inhibition has a greater effect. Targeting the PI3K pathway may be a useful approach to improve the chemotherapeutic sensitivity of cholangiocarcinoma.

## Background

Cholangiocarcinoma is a cancer characterized by early vascular invasion and metastasis. Patients with cholangiocarcinoma are often diagnosed at advanced stage. Threeyear survival rates of 35% to 50% can be achieved only in a subset of patients, who have negative histological margins at the time of surgery [1]. Palliative therapeutic approaches consisting of percutaneous and endoscopic biliary drainage have usually been used for these patients, since there is no effective chemotherapeutic treatment for this type of cancer [2]. A novel agent, oxaliplatin, has been extensively used as chemotherapeutic agent in treating solid tumors [3,4]. Oxaliplatin is a diaminocyclohexane platinum compound that acts like cisplatin to induce DNA adducts formation. Although early studies suggested that oxaliplatin might be used as an active agent against cholangiocarcinoma [5,6], more recent data indicated that cholangiocarcinoma cells were resistant to oxaliplatin [7]. Therefore, elucidating the mechanism of resistance to oxaliplatin in cholangiocarcinoma cells is crucial to improve the treatment of patients with advanced cholangiocarcinoma.

Activation of the phosphoinositide-3-kinase (PI3K)/Akt signaling pathway is frequently found in cholangiocarcinoma cells [8]. It has been suggested to be a key step leading to the resistance of cancer cells to chemotherapy, especially when using DNA-damaging agents such as cisplatin and oxaliplatin [9,10]. Furthermore, previous studies have demonstrated that PI3K/Akt activation regulates sensitivity of cells to G1 arrest induced by mTOR inhibitors [11]. Taken together, these data indicate that chemotherapeutic agents might function better in killing cancer cells if the PI3K pathway is blocked. In this study, we hypothesize that inhibition of PI3K or its downstream target, mTOR, may be increase oxaliplatin efficacy in treating cholangiocarcinoma. The effect of PI3K and mTOR inhibition on oxaliplatin sensitivity of cholangiocarcinoma cells is examined.

## Methods

### Cell culture and Materials

Ham's F12 medium and fetal bovine serum (FBS) were purchased from Gibco (Gibco, Grand Island, NY, USA). Polyclonal antibodies to Akt (phosphorylated at Ser473 and total), mTOR, PP70S6K and P38 MAPK (phosphorylated at Thr180/Tyr182 and total) were purchased from Cell Signaling (Cell Signaling Technology, Beverly, MA, USA). Oxaliplatin was purchased from Sanofi Aventis (Sanofi Aventis, Bridgewater, NJ, USA). Cell culture plastic plates were obtained from Nunc (Thermo Fisher, Rochester, NY, USA). LY294002 (PI3K inhibitor) was purchased from Calbiochem (EMD Chemicals, Gibbstown, NJ, USA). RAD001 (everolimus), an oral derivative of rapamycin, was generously provided by Novartis Pharma AG (Novartis International AG, Basel, Switzerland). Stock solutions (10 mmol/L) were dissolved in DMSO (Sigma-Aldrich, St. Louis, MO, USA), stored at -80°C, and diluted in fresh medium immediately before use.

The human intrahepatic cholangiocarcinoma cell lines RMCCA1 [12] and KKU100 (kindly provided by Dr. Banchob Sripa, Department of Pathology, Faculty of Medicine, Khon Kaen University) were grown in Ham's F12 medium supplemented with 10% FBS at 37°C in a 5% CO_2 _humidified atmosphere. For experiments, cells were grown in Ham's F12 medium supplemented with 1% FBS.

### Cell proliferation assay

For proliferation assay, cells were seeded in 96-well culture plastic plates at a density of 10,000 cells per well. Vehicle (PBS) or oxaliplatin in various concentrations (0–200 μM) were added to each well. For the Akt or mTOR inhibition studies, cells were treated with Vehicle (DMSO), LY294002 (PI3K inhibitor) or RAD001 (mTOR inhibitor), respectively, for 1 hour before the addition of oxaliplatin. Cells were then incubated for 48 hours before applying the WST-1 cell proliferation assay reagent (Roche Diagnostics, Laval, Quebec, Canada), according to the recommendation of the manufacturer. The amount of cell proliferation was assessed by determining the *A*_450 nm _of the cell culture media after addition of WST-1 for 2 hours. Results were reported as percentage of the inhibition of cell proliferation, where the optical density measured from vehicle-treated cells was considered to be 100% of proliferation. Percentage of inhibition of cell proliferation was calculated as follows: (1-*A*_exp group_/*A*_control_) × 100.

### Cell apoptosis assay

The number of apoptotic cells was determined with the Apo-BrdU TUNEL assay kit (Invitrogen, Carlsbad, CA, USA), following manufacturer's instructions. Briefly, cells were washed with cold PBS and then fixed with 1% paraformaldehyde and ice-cold 70% ethanol for 30 minutes. Fixed cells were labeled with BrdUTP using terminal deoxynucleotide transferase (TdT) at 37°C for 60 minutes and stained with Alexa Fluor 488-labeled anti-BrdU antibody for 30 minutes at room temperature. To score for apoptosis, cells were counterstained with DAPI, and at least 200 cells were counted under fluorescent microscope at 400× magnification. The percentage of apoptotic cells per experimental condition was then determined.

### Western blotting analyses

Approximately 500,000 cells were seeded in a six-well culture plate, followed by treatment with vehicle (PBS), or oxaliplatin for 12 hours. Cells were collected, washed with PBS and lysed in lysis buffer. Western blot analyses were performed as previously described [8]. The blots were first probed with antibodies against phospho-Akt, phospho-mTOR, phospho-P70S6K or cleaved caspase-3 and then reprobed with antibodies against total Akt, mTOR, P70S6K or caspase-3. Bound antibodies were detected using chemiluminescence.

### Statistical analysis

The experiments were all performed in triplicate, and each result is reported as the mean with SD. Data between three or more groups were compared using the one-way analysis of variance, followed by Dunnett's *post hoc *test. A *p*-value of less than 0.05 was considered statistically significant.

## Results

### Oxaliplatin slightly inhibits cholangiocarcinoma cell proliferation

**Figure 1:**
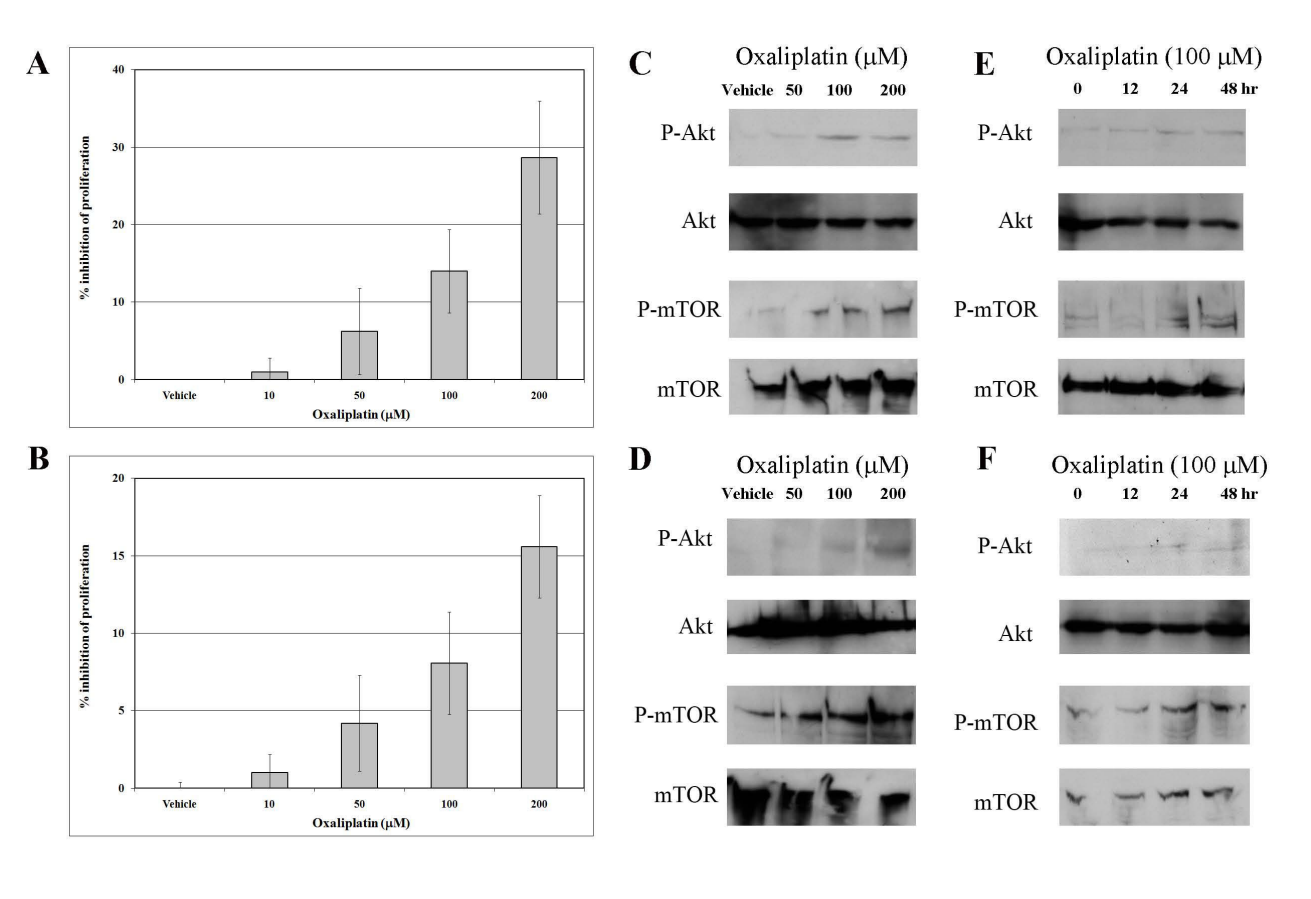
**Effect of oxaliplatin on cholangiocarcinoma cells**. (A) RMCCA1 and (B) KKU100 cells were treated with oxaliplatin at various concentrations (0, 10, 50, 100 and 200 μM) for 48 hours. Effect on cell proliferation was measured by WST-1 and analyzed by spectrophotometric analysis (Absorbance = 450 nm). Results are reported as percentage inhibition of cell proliferation, where the optical density value from vehicle-treated cells was set as 100% of proliferation. Results are represented by the mean ± SE of three independent experiments. Phosphorylation of Akt and mTOR in (C) RMCCA1 and (D) KKU100 cells following 0–200 μM oxaliplatin treatment for 12 h and phosphorylation of Akt and mTOR in (E) RMCCA1 and (F) KKU100 cells following 100 μM oxaliplatin treatments for 0–48 h were determined by western blotting. Total Akt and mTOR were used as loading control. Representatives of the three independent experiments are shown.

Cholangiocarcinoma cells were treated with 0–200 μM oxaliplatin for 48 hours, and then a cell proliferation assay was performed using WST-1. The percentage of cell proliferation inhibition was set at 0% when the cells were treated with vehicle (PBS). Both RMCCA1 and KKU100 displayed a slight dose-sensitivity to oxaliplatin. For RMCCA1, the inhibition of cell proliferation was 14.0% ± 6.54 and 28.7% ± 7.33 in cells treated with 100 and 200 μM of oxaliplatin, respectively. For KKU100, the inhibition of cell proliferation was 8.1% ± 3.31 and 15.6% ± 3.30 in cells treated with 100 and 200 μM of oxaliplatin, respectively (Figs. 1A and 1B).

### Phosphorylation of Akt and mTOR was induced by oxaliplatin in cholangiocarcinoma cells

Previous studies demonstrated that activation of PI3K pathway induced chemoresistance in cancer cells. To assess PI3K activation in cholangiocarcinoma cells after treatment with oxaliplatin, the levels of phosphorylated Akt and mTOR, two downstream signal transduction molecules in the PI3K pathway, were examined. Cholangiocarcinoma cells were treated with 0–200 μM of oxaliplatin for 12 hours or treated with 100 μM of oxaliplatin for 0–48 hours. Cells were then subjected to western blot analysis. The levels of Akt and mTOR phosphorylation increased as the concentration of oxaliplatin increased (Figs. 1C and 1D). In addition, the increase in the levels of phosphorylated Akt and mTOR is observed as early as 12 hours and as late as 48 hours after oxaliplatin treatment in both cell lines (Figs. 1E and 1F). This result is in agreement with that from a previous study, indicating that the mechanism of cell protection to chemotherapeutic agent is through the activation of the PI3K pathway [9,12].

### Inhibition of PI3K and mTOR increases the cytotoxicity of oxaliplatin in cholangiocarcinoma cell lines

**Figure 2:**
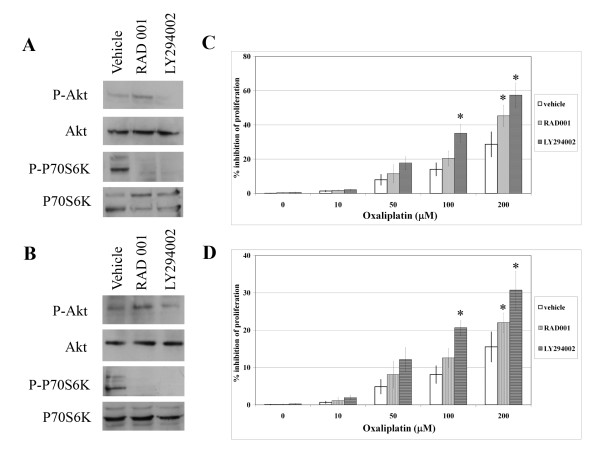
**Inhibition of Akt and mTOR increases the oxaliplatin-induced cytotoxicity in cholangiocarcinoma cell lines**. The effects of Akt (LY294002), and mTOR (RAD001) inhibitors on the phosphorylation of Akt and P70S6K in (A) RMCCA1 and (B) KKU100 cells were determined by western blotting. Total Akt and P70S6K were used as loading controls. Cells were treated with 10 μM LY294002, 0.5 μM RAD001 or control vehicle (DMSO) for 24 hours. Representatives from 3 independent experiments are shown. The effects of LY294002 and RAD001 in (C) RMCCA1 and (D) KKU100 cells following treatment with or without oxaliplatin are shown. Cells were treated with 10 μM LY294002, 0.5 μM RAD001 or control vehicle (DMSO), followed by the addition of 0–200 μM oxaliplatin for 48 hours. Cell proliferation was measured by WST-1 and analyzed by spectrophotometric analysis (Absorbance = 450 nm). Results are reported as a percentage inhibition of cell proliferation, where the optical density value from vehicle-treated cells were set as 100% of proliferation and represent the mean ± SE of three independent experiments. (*, *p *< 0.05 versus the same concentration of oxaliplatin)

To evaluate the effect of the PI3K pathway on oxaliplatin resistance, cholangiocarcinoma cells were treated with specific inhibitors of PI3K (LY294002) and mTOR (RAD001), with or without oxaliplatin. Western blot analysis was used to determine the levels of phosphorylation of Akt and P70S6K, the downstream targets of PI3K and mTOR, respectively. Cell growth was determined by the cell proliferation assay. When treated with LY294002, the cells clearly exhibit lower levels of Akt and P70S6K phosphorylation compared to what is seen under control conditions. RAD001 also significantly reduced the phosphorylation of P70S6K, but it increased the phosphorylation of Akt (Figs. 2A and 2B).

Oxaliplatin-induced resistance of cells was shown to be modulated by inhibitors of either Akt or mTOR. Cholangiocarcinoma cells were pretreated with either 10 μM LY294002 or 0.5 μM RAD001 for 1 hour, followed by incubation with 0–200 μM oxaliplatin. Pretreatment with LY294002 resulted in a two-fold increase in the percentage of inhibition of cell proliferation at both 100 and 200 μM of oxaliplatin when compared to the control (RMCCA1; from 14.1% ± 3.76 to 35.1% ± 5.14 at 100 μM oxaliplatin; *p *= 0.002 and from 28.6% ± 7.25 to 57.4% ± 7.19 at 200 μM oxaliplatin; *p *= 0.004 and KKU100; from 8.1% ± 2.33 to 20.7% ± 1.98 at 100 μM oxaliplatin; *p *= 0.09 and from 15.5% ± 4.02 to 30.7% ± 5.10 at 200 μM oxaliplatin; *p *= 0.01, (Figs. 2C and 2D). Pretreatment with RAD001 resulted in increased inhibition of cell proliferation only at high concentrations of oxaliplatin (RMCCA1; from 14.1% ± 3.76 to 20.5% ± 4.37 at 100 μM oxaliplatin; *p *= 0.2 and from 28.7% ± 7.25 to 45.3% ± 6.20 at 200 μM oxaliplatin; *p *= 0.04 and KKU100; from 8.1% ± 2.33 to 12.6% ± 2.54 at 100 μM oxaliplatin; *p *= 0.20 and from 15.5% ± 4.02 to 22.0% ± 2.58 at 200 μM oxaliplatin; *p *= 0.006). The significant increase of oxaliplatin-induced cytotoxicity in cholangiocarcinoma cells upon pretreatment with specific kinase inhibitors indicates that resistance of cholangiocarcinoma cells to chemotherapeutic agents can be modulated.

### LY294002 increases oxaliplatin-induced cell apoptosis

**Figure 3:**
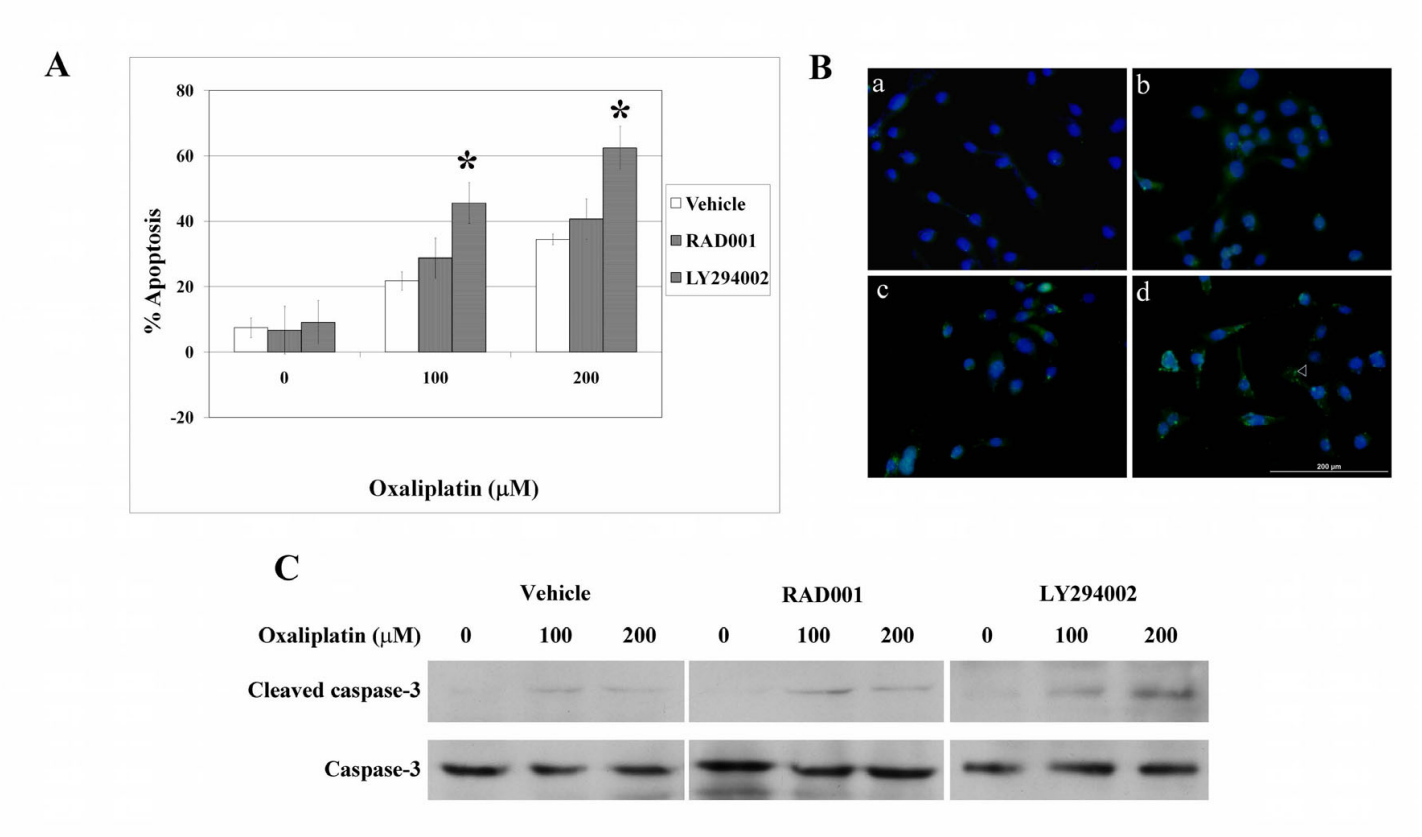
**LY294002 modulated oxaliplatin-induced cell apoptosis**. (A) Cells were added with 10 μM LY294002, 0.5 μM RAD001 or control vehicle and then treated with 0–200 μM oxaliplatin. TUNEL assay was done as described in the Methods section. The percentage of apoptotic cells in each group of treatments was demonstrated (values shown as mean ± SD, *; *p *< 0.05 versus the same concentration of oxaliplatin). (B) Apoptotic cells (green spots) were detected with an ApopTag Staining kit and counterstained with DAPI (blue). RMCCA1 cells were treated with Vehicle (a) and 100 μM oxaliplatin (b). Cells were treated with RAD001 plus 100 μM oxaliplatin (c) and with LY294002 plus 100 μM oxaliplatin (d) (Arrow head indicates the apoptotic cells). (C) Western blots analysis of cleaved caspase-3 and caspase-3 in each group of treatments. The blots were representative of three independent experiments.

In order to determine the mechanism by which LY294002 and RAD001 increase oxaliplatin-induced cytotoxicity, TUNEL apoptosis assays were performed. 10 μM LY294002, 0.5 μM RAD001 or control vehicle (DMSO) were added to RMCCA1 cholangiocarcinoma cells, followed by treatment of the cells with 0–200 μM oxaliplatin for 48 hours. Exposure to either LY294002 or RAD001 alone did not significantly alter the number of RMCCA1 apoptotic cells when compared to the control. However, the combination of LY294002 with 100–200 μM oxaliplatin significantly increased the number of apoptotic cells (from 21.8% ± 7.33 to 45.6% ± 6.13 at 100 μM oxaliplatin; *p *= 0.008 and from 34.5% ± 6.72 to 62.4% ± 6.68 at 200 μM oxaliplatin; *p *= 0.004). In contrast, the combination of RAD001 with 100–200 μM oxaliplatin did not significantly increase the number of apoptotic cells (Figs. 3A and 3B).

To verify that apoptosis was the direct cause of cell death, the presence of cleaved caspase-3, a central marker of apoptosis, was determined by western blot analysis. As shown in Fig. 3C, the level of cleaved caspase-3 was very low in cholangiocarcinoma cells treated with 10 μM of LY294002, 0.5 μM of RAD001 or oxaliplatin alone. On the other hand, the level of cleaved caspase-3 was increased in cholangiocarcinoma cells treated with LY294002 in combination with 100 or 200 μM of oxaliplatin.

## Discussion

Cholangiocarcinoma is a rapidly lethal disease and generally considered to be incurable. One of the main reasons for its low survival rate is that cholangiocarcinoma exhibits extensive local invasion and frequent regional lymph node metastasis. Most patients are not candidates for curative surgical resection [13]. Until recently, there has been no effective chemotherapeutic drug for this disease.

Oxaliplatin has been used for the treatment of a number of solid tumors including lung, gastric, and colorectal cancer [[3,6], and [14]]. Recently, a prospective multicenter phase II study focused on capecitabine and oxaliplatin (CAPOX) combination therapy in advanced cholangiocarcinoma [3,7]. Unfortunately, the results suggested that this regimen produced poor results for intrahepatic cholangiocarcinoma [7]. An alternative strategy is then needed to evaluate the efficacy of oxaliplatin as chemotherapeutic agent. We used two cholangiocarcinoma cell lines, RMCCA1 and KKU100, derived from cholangiocarcinoma patients to study the effect of oxaliplatin *in vitro*. These cell lines exhibited resistance to oxaliplatin, even at high concentrations (100–200 μM). In addition, we demonstrated that oxaliplatin-treated cholangiocarcinoma cells exhibit high levels of Akt and mTOR phosphorylation as a result of PI3K activation. Thus, we hypothesized that activation of the PI3K pathway in cholangiocarcinoma cells may, in turn, protect the cells from oxaliplatininduced cytotoxicity. Our results indeed showed that inhibition of Akt by LY294002 significantly increased oxaliplatin efficacy in inhibiting cell proliferation. This finding suggests that Akt phosphorylation might be attributed to oxaliplatin resistance in cholangiocarcinoma cells. This result is also consistent with recent evidence showing that the mechanism of drug resistance in cancer cells was primarily through the induction of PI3K/Akt pathways [15].

Previous studies demonstrated that exposure of cancer cells to oxaliplatin induced protein misfolding. These misfolded proteins are prone to oxidative stress as a result of better accessibility of reactive oxygen species (ROS) to the protein structure [16]. As a consequence, recruitment of Bax to the mitochondria, release of cytochrome c to the cytosol, activation of caspase-3 and apoptotic cell death take place in cancer cells treated with oxaliplatin. Recently, Kim et al. reported that the activation of Akt could inhibit oxaliplatininduced apoptosis through maintaining XIAP protein levels [10]. In this study, we demonstrate that inhibition of Akt by LY294002 increases the percentage of apoptotic cells after oxaliplatin treatment. In addition, activation of caspase-3 was clearly observed in cholangiocarcinoma cells treated with both LY294002 and oxaliplatin. These data indicate that activation of Akt in cholangiocarcinoma cells may be the key mechanism in inhibiting oxaliplatin-induced apoptosis.

PI3K and Akt regulate the processes of cellular glucose metabolism. Inactivation of PI3K and Akt may have deleterious effects on normal cell metabolism [17]. Therefore, only inhibitors of those downstream molecules of PI3K and Akt that are not involved in glucose metabolism should be considered for clinical treatment. The mammalian target of rapamycin is mTOR, a 289 kDa serine/threonine kinase. mTOR is a downstream effector of the PI3K/Akt signaling pathway involved in the regulation of many transduction processes of cell growth as well as cell cycle progression, membrane trafficking, protein degradation, and protein kinase C signaling and transcription [18].

Recently, a derivative of rapamycin, RAD001 (everolimus), has been developed. RAD001 has been shown to inhibit mTOR activity, thereby halting the proliferation of cancer cells, both *in vitro *and *in vivo*. Phase II clinical trials with RAD001 are currently being performed for many types of cancers [18,19]. Based on the results of our study, the 0.5 μM RAD001 alone did not inhibit the proliferation of cholangiocarcinoma cells. This is consistent with a previous study, which demonstrated that RAD001 has only cytostatic effects in cancer cells. To induce cytotoxicity of RAD0001 in cancer cells, other chemotherapeutic drugs should be combined with RAD0001 [18,20]. For example, pretreating ovarian cancer cells with RAD001 can increase their sensitivity to cisplatin [21]. In this study, we found that RMCCA1 and KKU100 displayed high levels of Akt and mTOR phosphorylation after treatment with oxaliplatin. Pretreatment of cholangiocarcinoma cells with 0.5 μM RAD001 significantly increased the sensitivity of oxaliplatin when used at 200 μM. However, pretreatment with 0.5 μM RAD001 did not significantly increase the efficacy of oxaliplatin when used at 100 μM. In addition, the number of apoptotic cells and the activation of caspase-3 did not significantly increase when the cells were exposed to both RAD001 and oxaliplatin. This might be explained by the fact that inhibition of P70S6K by RAD001 induces IGF-IR/IRS-1/PI3K signaling, eventually increasing the level of Akt phosphorylation [22]. This feedback mechanism might be responsible for the decrease in sensitivity to oxaliplatin, leading to a reduction in the inhibition of cell proliferation. These results are consistent with the recent report that inhibition of mTOR resulted in Akt activation in several human cancer cell lines [22].

In summary, this study presents the possible mechanism in oxaliplatin resistance in cholangiocarcinoma cells. As proof-of-concept, we are able to show that activation of the Akt signaling pathway has a potent effect on oxaliplatin resistance. The model presented here may serve as a practical tool for identifying the molecular mechanism of chemotherapeutic drug-resistance in cholangiocarcinoma cells.

## Abbreviations

mTOR: Mammalian target of rapamycin; PI3-K: Phosphatidylinositol-3 kinase; TUNEL: Terminal deoxynucleotidyl transferase.

## Competing interests

The authors declare that they have no competing interests.

## Authors' contributions

KL conceived of the study, designed, coordinated the study, statistical analysis and drafted the manuscript, SN carried out the proliferation and western blotting assays and helped with the statistical analysis, WU carried out the TUNEL and western blotting assays, SL revised the manuscript critically for important intellectual content, and helped draft the manuscript, ST also gave final approval for the paper to be submitted for publication.
